# Public Perception of the Fifth Generation of Cellular Networks (5G) on Social Media

**DOI:** 10.3389/fdata.2021.640868

**Published:** 2021-06-18

**Authors:** Kia Dashtipour, William Taylor, Shuja Ansari, Mandar Gogate, Adnan Zahid, Yusuf Sambo, Amir Hussain, Qammer H. Abbasi, Muhammad Ali Imran

**Affiliations:** ^1^ James Watt School of Engineering, University of Glasgow, Glasgow, United Kingdom; ^2^ School of Computing, Edinburgh Napier University, Edinburgh, United Kingdom; ^3^ Queen Mary University of London, London, United Kingdom; ^4^ Artificial Intelligence Research Center (AIRC), Ajman University, Ajman, United Arab Emirates

**Keywords:** sentiment analysis, 5G, mobile network quality, machine learning, opinion mining

## Abstract

With the advancement of social media networks, there are lots of unlabeled reviews available online, therefore it is necessarily to develop automatic tools to classify these types of reviews. To utilize these reviews for user perception, there is a need for automated tools that can process online user data. In this paper, a sentiment analysis framework has been proposed to identify people’s perception towards mobile networks. The proposed framework consists of three basic steps: preprocessing, feature selection, and applying different machine learning algorithms. The performance of the framework has taken into account different feature combinations. The simulation results show that the best performance is by integrating unigram, bigram, and trigram features.

## 1 Introduction

The fifth generation (5G) mobile network is the newest global wireless standard after 1G, 2G, 3G, and 4G. The 5G network is a new type of network which is designed and developed to connect virtually everyone and everything together and consists of different machines, objectives, and devices. In addition, 5G is required to be more efficient and economical in terms of key performance indicators (KPIs). KPIs are of interest to stakeholders and different applications. These KPIs from an operator perspective, consist of capacity, reliability, and quality of service. From the user perspective, the KPIs include uninterrupted connection, infinite capacity, and zero latency. However, no technology can offer infinite capacity or zero latency. In the past few years, there has been lots of research carried out on the next generation mobile network, which consists of different opportunities and challenges. The challenges of 5G have been discussed in different literature. The most significant of these are ultra-dense networks and millimeter waves, however, there are other technologies which are significant for the next generation network such as two-layer architecture and cognitive radio-based architectures which have great performance. In the 5G network, network data analytic and machine learning systems can perform a key role. The technique to understand people’s behavior towards the 5G network is vital and it helps to improve the performance of network communication OPINCARIU et al. (2019), Sharma et al. (2020).

With the advent of social media and e-commerce, websites allow users to share opinions and feedback about different products and services. Customers can make important decisions by reading other people’s experiences. In addition, customer feedback can be classified in order to make improvements on the service or product. For example, if a person wants to buy a mobile phone and the reviews provide negative information related to the battery, operation speed, or camera, this can influence the consumer’s decision. In addition, this can assist in providing better mobile quality by taking into account the complaints made by past customers and making informed improvements to products. As another example, if someone wants to book a hotel, the buyer can look over the online reviews to understand previous customer experiences such as cleanliness and services for the hotel. However, there are billions of bytes of data generated per day consisting of user feedback which cannot be manually labeled and analyzed for individual organizations and companies Yadav and Vishwakarma (2020). Sentiment analysis is the process of automatically understanding and classifying the data into positive and negative information from the source material such as reviews and comments. The main task of sentiment analysis is to assign polarity into sentences (positive or negative). However, the online review is a mixture of positive and negative comments about different aspects of the products or services instead of expressing positive or negative opinions. For example, “the 5G mobile network is extremely fast, however I do not feel secure while I am using it.” The sentence expresses positive sentiment towards the speed of the network and negative sentiment towards the security of the network Kumar and Jaiswal (2020), Dashtipour et al. (2020).

Most of the current sentiment analysis approaches focus on analyzing products and movie reviews, and there is less work been carried out in different fields such as people’s perception towards 5G. However, most of the current approaches consider a small corpus, which makes the task difficult for machine learning approaches to identify the overall polarity of a sentence. Furthermore, current review sentences consist of lots of sarcastic and ironic words which make it difficult to determine the overall polarity for these types of sentences. For example, “tell me something I don’t know.” Most of the current approaches for sentiment analysis fail to understand that real noisy text consists of sarcasm, idioms, informal words, and sentences with spelling mistakes. In addition, there is scarce availability of tools and resources. Lexicon and labeled corpus are some of the tools which are available for sentiment analysis. This limited range of available tools is the main bottleneck in the design of sentiment analysis approaches Kaity and Balakrishnan (2019). One of the main issues for sentiment analysis approaches is the lack of a labeled dataset. However, it is worth mentioning that, there are lots of unlabeled datasets available online, but it is time consuming for users to manually label these datasets.

In order to address the aforementioned issues and limitations, a framework that exploits n-gram features has been proposed to identify the polarity of sentences. This proposed approach demonstrated the overall performance and effectiveness of polarity detection in real noisy data. The n-gram features are based on the linguistic text rules that allow researchers to extract text features from sentences. As a result, the n-gram features take into account the relation between keywords and the word order and individual word polarity to determine the underlying polarity of the sentence. We perform an extensive and comprehensive set of experiments using novel corpus and compare the performance of the approach with different selected features. The support vector machine (SVM), logistic regression, naive Bayes, and multilayer perceptron (MLP) algorithms were used to evaluate the performance of the approach. The comparative simulation results show that the proposed approach achieved better performance as compared to the state-of-the-art approach.

The rest of the paper is organized as follows: In *Related Work*, related work is presented, *Methodology* presents the proposed framework, *Experimental Results* presents the experimental results, *Discussion* presents the discussion, and finally *Conclusion* concludes the work and presents the future work.

## 2 Related Work

Extensive research in the current literature shows that machine learning has been used in different fields such as sentiment analysis Dashtipour et al. (2016a), Dashtipour et al. (2016b), Dashtipour et al. (2017a), Dashtipour et al. (2017b), Gogate et al. (2017b), Dashtipour et al. (2017c), Shiva et al. (2017), Gogate et al. (2017a), Gogate et al. (2018), Dashtipour et al. (2018), Dashtipour et al. (2019), cyber-security Adeel et al. (2019a); Jiang et al. (2019), Gogate et al. (2019b), Gogate et al. (2019a), Adeel et al. (2019b), Ozturk et al. (2019), Jiang et al. (2020), Dashtipour et al. (2020); speech enhancement Gogate et al. (2020a), Gogate et al. (2020b), Liaqat et al. (2020), Taylor et al. (2020), Liaqat et al. (2020), Adeel et al. (2020), Guellil et al. (2021), Hussain et al. (2021), Dashtipour et al. (2021), hand-written recognition Ahmed et al. (2021), and posture detection; Liaqat et al. (2021), etc., However, research has not been carried out to detect sentiment polarity for tweets related to 5G.

In the literature, extensive research has been undertaken to implement different sentiment analysis approaches. Microblogging websites are the biggest platform that allows users to share their thoughts and opinions in the public domain. Twitter is the most well-known microblogging website that allows people to express their feelings and emotions in the form of “tweets” with a character range of 280. There are over 250 million tweets expressing the feelings and emotions of people with different opinions and situations Duong et al. (2019), Mamgain et al. (2016).

Twitter users vary from politicians to everyday people that provide different types of reviews from different points of views. This is the main reason a dataset collected from Twitter is used in this paper. There is lots of research carried out in this domain to determine people’s perception towards different products such as Sony mobiles. For example, Sharma et al. (2016) attempted to find the most well-known smartphones in India, tweets were collected and then machine learning was applied to determine a brand reputation score. This was done to help customers find the most branded smartphones in India. Somula et al. (2020) proposed an approach to perform a sentiment analysis to determine the winner of the US election in 2016, the tweets were collected if they mentioned Donald Trump and Hillary Clinton, the analysis of the tweets revealed that Donald Trump received more positive scores as compared to Hillary Clinton. Mehta et al. (2020) proposed a sentiment analysis approach to identify the best Indian airlines through Twitter, the analysis of results revealed that the customers were happier with Air India services compared to SpiceJet.

Kumari and Haider (2020) proposed an approach using the Twitter API to collect corpus. After pre-processing and use of natural language processing, the hybrid classifier utilized machine learning and long short-term memory to improve the performance of the approach. The experimental results revealed that the proposed model achieved better performance as compared with state-of-the-art approaches. Usama et al. (2019) introduced a novel model multilevel feature extraction and feature combination by using a convolutional neural network (CNN) and recurrent neural network (RNN) to identify the sentiment in movie reviews. The CNN and RNN received sentiment text as input and learnt different features to network architecture. The word embedding fed into the CNN and learnt multilevel contextual features from every layer of the CNN and performed multilevel features fusion. Finally, the multilevel and multitype features were combined and a softmax classifier was used to identify the final polarity of the sentences. Most of the aforementioned studies implement a lexicon to determine the polarity of the text. However, the use of a lexicon fails to identify the polarity of word order in the sentence. In addition, most of the current studies use rules to detect negation in a sentence which cannot be directly applied to the English language. However, we need a framework to identify the polarity of the sentence without developing a lexicon which is time consuming. Therefore, we implemented a framework for Twitter sentiment analysis that integrates feature engineering and machine learning to improve the performance and robustness of polarity detection in real noisy data.

## 3 Methodology

**FIGURE 1:**
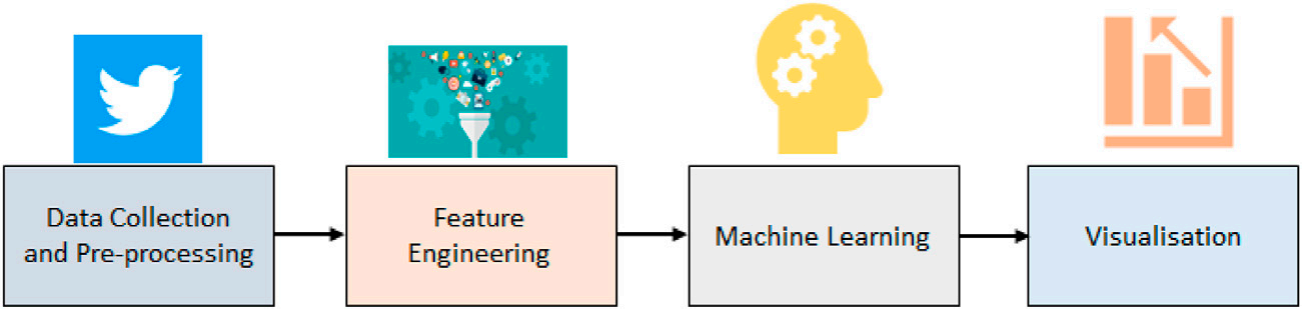
Proposed framework for sentiment analysis for people’s perception of the fifth generation of cellular networks (5G).

Figure 1 This section describes our proposed novel context-aware framework for 5G sentiment analysis. The proposed framework more accurately exploits the polarity of sentences when compared to traditional word occurrence frequency-based approaches.

Data Collection: In order to collect data, we use the Twitter API to collect data related to 5G in the United Kingdom. The data were collected from January 2018 until August 2020. Table 1 shows the related keywords for the fifth generation of cellular networks (5G).

**TABLE 1:** Next generation mobile network (5G).

Keywords
5G
Next generation mobile network
Fifth generation of technology
5G devices

Pre-processing: The tweets corpus is collected using the Twitter API and labeled as positive and negative using SentiWordNet. The corpus is divided into a training set (60%), test set (30%), and validation set (10%) to apply machine learning algorithms including SVM, naive Bayes, and MLP. The corpus is tokenized and normalized. The tokenization technique is used to break sentences into words. For example, “I really like mobile” will be converted into words such as “I,” “really,” “like,” and “mobile.” Afterwards, the normalization technique is used to normalize the tweets. For example, “The speed for 5G mobile is gr8” will be converted into “The speed for 5G mobile is great.”

N-gram: N-gram features are widely used in the different approaches of sentiment analysis. When one term is taken the feature is called unigram, for two terms it is called bigram, and three terms are called trigram. In our proposed approach we use unigram, bigram, trigram, and a combination of n-gram features.

SentiWordNet: We use SentiWordNet, which is a widely available online lexicon, to assign sentiment polarity (−1, 0, 1) to sentences.

Machine learning classifiers: In order to evaluate the performance of the approach, the machine learning classifier is used to evaluate the performance of the approach. The scikit-learn python package is used to develop the multilayer perceptron (MLP), logistic regression, linear SVM, RBF SVM algorithms, and naive Bayes is used to train the model. The MLP consists of one hidden layer which can be applied to supervised problems, the MLP is a set of inputs and outputs and it learns to model the correlation between input and output. However, the main issue with MLP is that it consists of many parameters which are fully connected and each node is connected to another node which can result in redundancy and inefficiency. The main advantage of naïve Bayes is that it performs quickly and save lots of time; however, the prediction can be wrong. In addition, the main advantage of logistic regression is how easy it is to implement and train; however, the number of observations is less than number of features. Finally, the main advantages of SVM is that it works work well with unstructured data such as text, as the tweets are unstructured, therefore, the SVM performs well with tweets data. However, the main issue with SVM is that choosing the correct kernel is not easy.

## 4 Experimental Results

In order to calculate the sentiment polarity of the sentence, SentiWordNet has been used to calculate the overall polarity of the tweets. The tweets are extracted using different keywords such as “5G,” “next generation mobile network,” “fifth generation of technology,” and “5G devices.” The tweets are collected. After tweet collection, the punctation and stop words are removed and sentence are normalized, and then they are converted into bag of words (BOWs) and finally machine learning classifiers are trained. In the pre-processing stage, the sentences are normalized, for example, the word “fishing” is converted into “fish.”

In order to evaluate the performance of the proposed approach, the tweets are converted into BOWs. These BOWs are sent to machine learning algorithms including linear and RBF SVM, naive Bayes, logistic regression, and MLP to evaluate the performance of the approach. The initial experimental results demonstrate that the combination of unigram and bigram achieved accuracy of 86.71%.

Dataset: In order to evaluate the performance of the approach, the 5G hashtag is used to collect more than 50,000 tweets, and the positive and negative polarity is assigned to the tweets using the SentiWordNet lexicon. Neural tweets are eliminated. The experimental results show that the combination of unigram and bigram achieved a better performance when compared to other approaches. In order to evaluate the performance of the proposed approach, different evaluation metrics including accuracy, precision, recall, and f-measure are used:

$$Precision=\frac{TP}{TP+FP}$$ (1)

$$Recall=\frac{TP}{TP+FN}$$ (2)

$$F\_measure=2\text{*}\frac{Precision\text{*}Recall}{Precision+Recall}$$ (3)

$$Accuracy=\frac{TP+TN}{TP+TN+FP+FN}$$ (4)

where TP denotes true positive, TN presents true negative, FP is false positive, and FN represents false negative. In addition, Table 2 shows the parameters that are used to trained the machine learning methods. The scikit-learn package is used to train the machine learning classifiers. In addition, the training time for each model is presented in Table 2.

**TABLE 2:** Parameters of ML algorithms.

Algorithm	Parameter	Time
SVM	RBF kernel	4 min and 21 s
Naive Bayes	Sample weight = none	2 min and 12 s
MLP	Activation = relu	3 min and 31 s
Logistic regression	Penalty = l2	3 min and 42 s

Table 3 shows the results of different N-gram features. The unigram (Uni), bigram (Bi), and trigram (Tri) is extracted from the sentence, as the comparative experimental result shows the unigram (Uni) achieved a better performance when compared to other features.

**TABLE 3:** Results of N-gram features.

Feature	Classifier	Accuracy	Precision	Recall	F-score
Uni	MLP	85.92	0.86	0.86	0.86
Uni	LR	86.14	0.86	0.86	0.86
Uni	Linear SVM	84.79	0.84	0.84	0.84
Uni	RBF SVM	62.61	0.72	0.63	0.54
Uni	NB	86.48	0.87	0.86	0.87
Bi	MLP	79.16	0.80	0.79	0.78
Bi	LR	78.37	0.79	0.78	0.78
Bi	Linear SVM	73.98	0.78	0.74	0.72
Bi	RBF SVM	62.61	0.72	0.63	0.54
Bi	NB	78.82	0.80	0.79	0.78
Tri	MLP	73.42	0.77	0.73	0.71
Tri	LR	72.52	0.77	0.73	0.70
Tri	Linear SVM	69.48	0.77	0.69	0.65
Tri	RBF SVM	62.61	0.72	0.63	0.54
Tri	NB	71.39	0.77	0.71	0.68

Table 4 shows the comparison of different N-gram features. The empirical results show that the combination of unigram (Uni) and bigram (Bi) achieved a better performance when compared to other features.

**TABLE 4:** Comparison of combination of N-gram features.

Feature	Classifier	Accuracy	Precision	Recall	F-score
Uni + Bi	MLP	86.71	0.87	0.87	0.87
Uni + Bi	LR	86.14	0.86	0.86	0.86
Uni + Bi	Linear SVM	85.92	0.86	0.86	0.86
Uni + Bi	RBF SVM	62.61	0.72	0.63	0.54
Uni + Bi	NB	85.81	0.86	0.86	0.86
Uni + Tri	MLP	85.13	0.85	0.85	0.85
Uni + Tri	LR	86.59	0.87	0.87	0.87
Uni + Tri	Linear SVM	86.48	0.87	0.86	0.87
Uni + Tri	RBF SVM	62.61	0.72	0.63	0.54
Uni + Tri	NB	85.47	0.85	0.85	0.85
Bi + Tri	MLP	76.91	0.79	0.77	0.76
Bi + Tri	LR	77.02	0.79	0.77	0.76
Bi + Tri	Linear SVM	77.02	0.80	0.77	0.76
Bi + Tri	RBF SVM	62.01	0.60	0.59	0.6
Bi + Tri	NB	71.28	0.70	0.69	0.70

**FIGURE 2:**
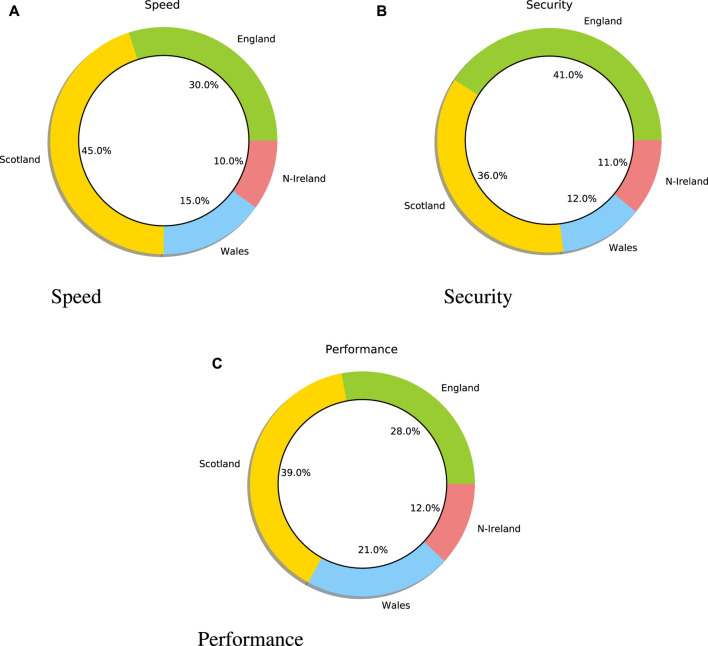
Positive trends towards the fifth generation of cellular networks (5G).

Figure 2 displays the positive sentiment towards fifth generation of cellular networks (5G) technology, and the speed, security, and performance of 5G. For example, the user has a positive opinion of speed, security and performance. In order to find the most positive keywords towards 5G, the frequency of positive words in a sentence is calculated. The word frequency shows that the most discussed keywords are speed, security, and performance. As shown in Figure 2 1), most positive tweets about speed are from Scotland and England. For example, 45% of tweets in Scotland and 30% in England have a positive opinion about the speed of 5G. Comparatively, Figure 2 2) demonstrates that most positive tweets about security are also from England and Scotland. A total of 41% of tweets in England and 36% in Scotland include a positive opinion about the security of 5G. Figure 2 3) shows that the most positive tweets about performance come from Scotland and England; 39% of tweets in Scotland and 28% in England include a positive opinion about the performance of 5G.

**FIGURE 3:**
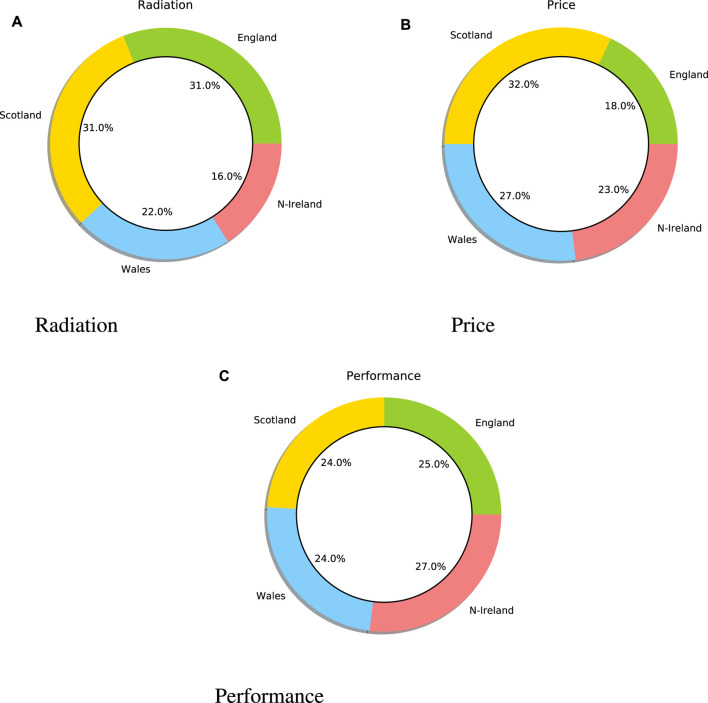
Negative trends towards the fifth generation of cellular networks (5G).

Figure 3 displays the negative sentiment towards fifth generation of cellular networks (5G) technology, including the radiation, price, and poor performance of 5G. For example, most users are concerned that the 5G signal might cause cancer, that the price of a 5G mobile is very expensive, and that the performance of 5G mobiles may be poor. In order to find the most negative keywords associated with 5G, the frequency of the negative words in a sentence is calculated. The word frequency shows that the most discussed keywords are radiation, price, and performance. As Figure 3 1) shows most Twitter users who are located in England and Scotland believe that 5G can cause cancer. It is to be noted that 31% of tweets in Scotland and 31% in England include negative concerns about the cause of radiation using 5G. In addition, Figure 3 2) shows that most Twitter users who are located in Scotland believe that the price of a 5G mobile is very expensive. It is worth mentioning that 32% of tweets in Scotland and 27% of tweets in Wales include negative concerns about the expensive price of 5G technology. Additionally, Figure 3 3) shows that most Twitter users in Northern Ireland are less satisfied with the current performance of 5G mobiles. It is to be noted that 27% of tweets in Northern Ireland and 25% of tweets in England include negative concerns about the current performance of 5G devices.

**FIGURE 4:**
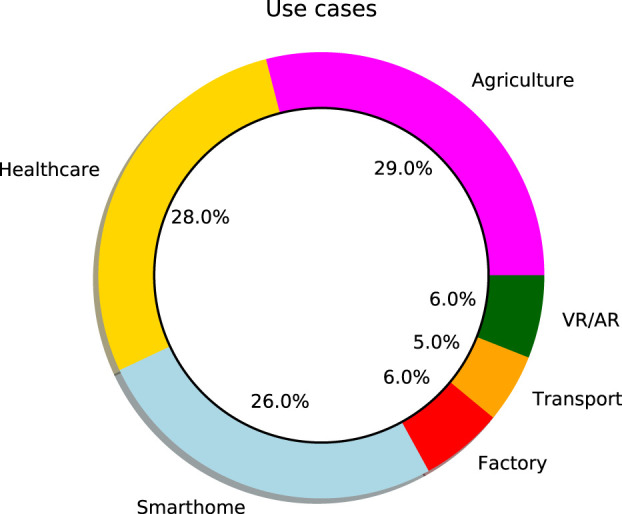
Trend of United Kingdom towards the fifth generation of cellular networks (5G).

In contrast, Figure 4 displays the overall trends for Twitter users. The most discussed trends include agriculture (29%), healthcare (28%), and smarthome (26%).

**FIGURE 5:**
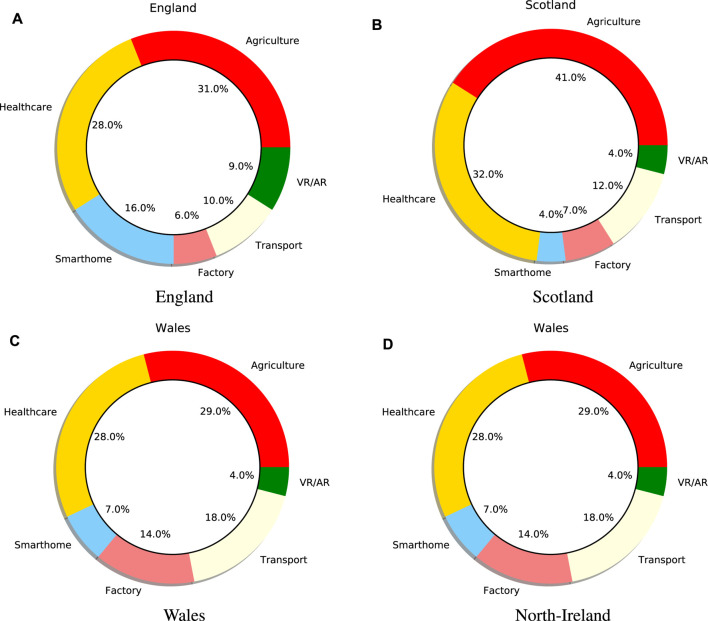
Most discussed trends for the fifth generation of cellular networks (5G).

Figure 5 displays the most discussed trends pertaining to 5G technology, including the radiation, price, and poor performance of 5G. For example, most users are concerned that the 5G signal might cause cancer, the price of the 5G mobile which is very expensive, and also the low performance of 5G mobiles. As shown in Figure 5, in England 1) the most discussed trends are agriculture (31%), healthcare (28%), and smarthome (16%). In addition, in Scotland 2) the most discussed trends are agriculture (41%), healthcare (32%), and transport (12%). Additionally, in Wales the most discussed trends are agriculture (29%), healthcare (28%), and transport (18%). Finally, in Northern Ireland, the most discussed trends are agriculture (29%), healthcare (28%), and transport (18%).

**FIGURE 6:**
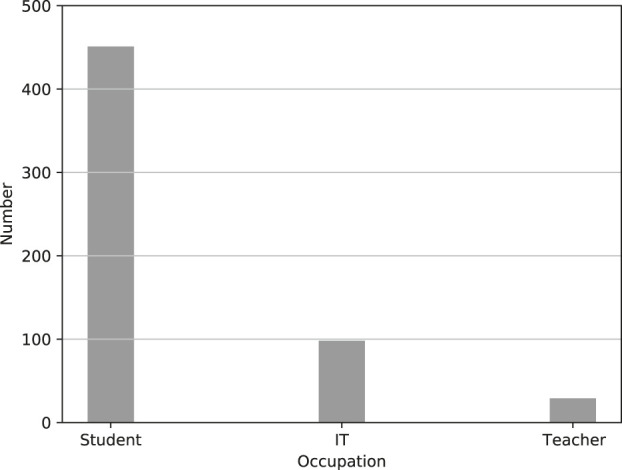
Occupation of Twitter users most positive towards the fifth generation of cellular networks (5G).

Figure 6 displays the occupation of the Twitter users who had the most positive comments aboutthe fifth generation of cellular networks (5G).

**FIGURE 7:**
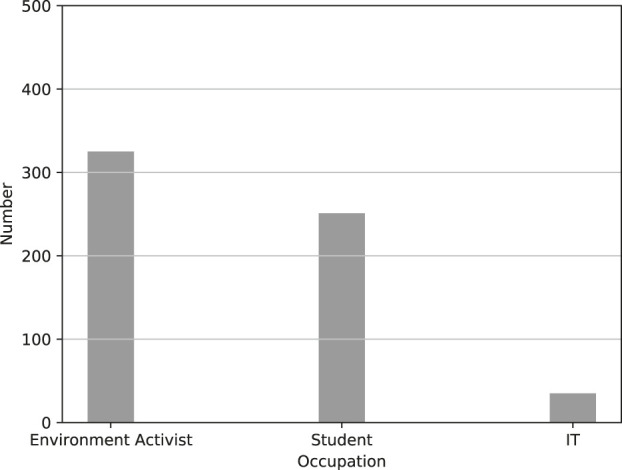
Occupation for Twitter users most negative towards the fifth generation of cellular networks (5G).

Figure 7 displays the occupation of the Twitter users who had the most negative comments about the fifth generation of cellular networks (5G).

As shown in Table 5, the top 10 positive and negative bigram keywords related to 5G are presented. Tweets concerning the price of 5G network generation are positive and concerning EU countries are negative.

**TABLE 5:** Most frequent positive bigrams for next generation mobile network (5G).

Positive bigram	Negative bigram
5G cheap	Low performance
Good coverage	Awful services
Good supply	Blow Huawei
Great performance	Hate 5G
High security	Low speed
Fast speed	Low coverage
Great system	Low frequency
Communication networks	5G crap
Nokia performs	Slow 5G
Creat satisfaction	Expensive technology

As shown in Table 6, the top 10 positive and negative trigram keywords related to 5G are presented. Tweets concerning the price of 5G network generation are positive and concerning EU countries are negative.

**TABLE 6:** Most frequent positive trigrams for next generation mobile network (5G).

Positive trigram	Negative trigram
Cheap 5G phones	Slow coverage 5G
Good coverage phone	Low suppliers services
Cest 5G phones	Blow Huawei China
Good Supply 5G	5G slow connection
Great areas coverage	5G low frequency
Good test zones	About 5G dangers
5G fast speed	Low frequency 5G
5G good communication	New 5G crap
Great 5G signal	Expect 5G slow
Nokia performs well	Expensive new technology

## 5. Discussion

In this study, the tweets related to the next generation mobile network (5G) were analyzed and their sentiment polarity was identified. The sentiment analysis of 5G keywords in tweets were considered because these tweets carried lots of information related to 5G. Only English tweets were considered because it that language has a large coverage and is the most widely used common language in the world. In the current study, more than 10,000 tweets were retrieved. There were many duplicate tweets in the retrieved data which were removed from the database. However, we did not limit our dataset to the tweet discussion of online users, we used news feeds related to 5G network communication for analysis because the news consisted of valuable information related to incidents.

Moreover, as part of the data pre-processing we removed the emoji characters from the tweets. However, it is worth mentioning that emoji characters are commonly used in tweets and they are useful for identifying the overall polarity of the tweets. In future work, we intend to consider emoji characters as part of the process to identify the overall polarity of tweets as emoji characters can provide more accurate sentiment scores. In contrast, the time frame to analyze the specific tweets related to 5G might be associated with events which increased the number of tweets on certain dates, weeks, or months. It is worth mentioning that there were lots of tweets related to conspiracy theories, for example some of tweets believed that 5G could cause COVID-19 in people.

Herein we explain the most popular topics discussed which can change the overall sentiment polarity of the tweets into positive:

Speed: The most discussed topic related to 5G was speed of the current network. Most of the tweets were positive towards the speed of 5G. However, it is to be noted that a large number of tweets were still negative about the bandwidth and speed of the 5G mobile network.

Security: In addition, another highly discussed topic related to 5G was the security of 5G services. Most of the Twitter users were positive towards the current security of the services for 5G. For example, “5G is more secure than 4G.”

Performance: Additionally, the performance of the 5G mobile network was discussed in most of the tweets. Most of the tweets were positive towards the performance of 5G network communication. For example, “I am really excited [about the] current performance of [the] 5G mobile.”

Herein we explain the most popular topics discussed which can change the overall sentiment polarity of the tweets into negative:

5G expose users to cancer: Our findings show that most of the tweets were worried that the next generation mobile network can cause cancer. For example, “I won’t buy [a] 5G mobile, because it can cause cancer.” There were strong negative comments towards the health issues of 5G network communication. Most of the Twitter users believed that 5G could cause damage to their health.

5G price: Our analysis shows that most of the Twitter users felt negatively towards the price of 5G mobiles. For example, one tweet was particularly clear: “How can I afford to buy such expensive mobiles.”

Speed: Furthermore, one of the most discussed topics concerning Twitter users was the speed of the 5G mobile network. For example, “I recently bought [a] 5G mobile but I do not like the speed.”

Table 7 shows examples of positive and negative tweets towards the fifth generation of cellular networks (5G).

**TABLE 7:** Examples of positive and negative tweets towards the next generation mobile network (5G).

Positive	Negative
Currently, the 5G phones are the best that you can buy right now	With 5G as dangerous to our health
5G has great performance	5G will be our deaths cancer rates will skyrocket
I gotta say, the vivo nex 3 5G has a pretty great DAC	5G is horrific, it causes cancer, it’s already banned in some countries
The global economic potential of 5G is staggering. It is predicted to add up to 3 million new jobs and create $500 billion	It’s not good enough, the 5G privacy
I Had good experience with 5G	5G is no good for human health

## 6 Conclusion

In this study, we performed a series of sentiment analyses on data retrieved from Twitter. The Twitter data under investigation were related to the fifth generation of cellular networks (5G). We collected relevant tweets in the English language. Therefore, we proposed a framework for mobile networks (such as 5G) based on different feature combinations. The performance of the proposed framework was evaluated using different feature combination in terms of different evaluation metrics such as accuracy, precision, recall, and f-measure. In addition, we compared the proposed method with different machine learning algorithms such as naïve Bayes, MLP, and SVM, etc., In addition, we analyzed the tweets to understand user perception of 5G. As part of our future work, we intend to extend the current framework for multilingual sentiment analysis and integration of a closed loop self organizing network algorithm with the proposed user sentiment analysis framework.

## Data Availability

The raw data supporting the conclusion of this article will be made available by the authors, without undue reservation.
